# Influence of mate and nest-site fidelity on a declining, urban avian population

**DOI:** 10.1371/journal.pone.0326160

**Published:** 2025-06-25

**Authors:** Lynne A. Trulio, Debra A. Chromczak, Philip G. Higgins

**Affiliations:** 1 Department of Environmental Studies, San José State University, San Jose, California, United States of America; 2 Environmental Researcher and Consultant, Riegelsville, Pennsylvania, United States of America; 3 Talon Ecological Research Group, San Jose, California, United States of America; Tshwane University of Technology, SOUTH AFRICA

## Abstract

As urbanization reduces species’ habitats and population sizes, managers need information on whether within-population processes, such as changes in mate and nest-site fidelity and dispersal distances, may be contributing to declines. Few avian studies have examined changes in these behaviors in declining populations or in urban settings. We investigated whether mate fidelity, nest-site fidelity or breeding dispersal distance changed over time in a population of burrowing owls (*Athene cunicularia*), a short-lived, socially-monogamous species. During the 18-year period of the study, the population declined by 69% in urban Santa Clara County, California, USA--a region of rapid urbanization. We assessed whether these behaviors were influenced by key factors including age, breeding success in the previous year, and years with the same mate, and examined the relationship between mate and nest-site fidelity over time and annual reproductive success. Our analyses showed no change over time in mate fidelity rates, nest-site fidelity rates, dispersal distances or annual reproductive success, indicating these behaviors remained stable even during a severe population decline. Although burrowing owls are a short-lived species, we found that increasing years with the same mate resulted in increased nest-site fidelity and annual reproductive success. To achieve increasing annual reproductive success in this species and others with similar fidelity behaviors, nest sites and pairs must be protected over many years allowing mates to stay together in the same nest territory. Since these fidelity behaviors were maintained during the population decline, other factors require investigation to determine the causes for decreases in this population. Burrowing owls are an urban-adaptable species that can maintain important fidelity behaviors in human-altered habitats. However, even such species are subject to population declines in urban settings.

## Introduction

Urbanization is expanding world-wide, with projections that urban land cover will quadruple between 2000 and 2050 [1]. Some animals respond to urban settings in ways that allow them to adapt, such as through behavioral plasticity, while others are excluded from these human-altered settings [2,3]. Species negatively affected by urbanization may experience disruptions to reproduction that reduce or eliminate populations [2,4]. Mate fidelity and nest-site fidelity (and its inverse, breeding dispersal) are behaviors that influence reproduction, breeding performance and population dynamics [5–7] and are especially supported in stable habitats where nest sites are able to persist from year to year [5,8]. Thus, fidelity patterns can be disrupted in unstable areas experiencing rapid change, such as occurs with urbanization [4].

Species living in urban settings can experience conditions that could change mating and/or nest site fidelity. Such conditions may include anthropogenic noise that masks or disrupts calls/songs necessary for finding mates [9–11] and nest site disturbance due to urban activities that disrupt nest-site fidelity [12,13]. Habitat fragmentation, a typical impact of urbanization [14,15] can isolate populations, thereby inhibiting breeding dispersal and potentially leading to excessive levels of philopatry, detrimentally long pair bonds, and inbreeding [16]. Merkle et al. (2022) [17] propose that, in some circumstances, site fidelity may become a maladaptive trait for populations exposed to rapidly-changing, human-altered environments. In such situations, populations adhering to site fidelity in the face of rapid environmental change may exhibit lower fitness than populations in undisturbed habitats [17]. Despite these potential effects of anthropogenic habitat change on species behavior, the effect of urban impacts on mating systems in animal populations has not been well studied [14]. Understanding the potential role of changes to mate and/or nest-site fidelity in declining populations may help managers preserve species in the face of urbanization.

Mate fidelity, remaining with the same mate for multiple consecutive breeding seasons, is expected to confer fitness advantages through familiarity with mates, thereby leading to increased breeding success [7,18]. A number studies have shown that pairs of birds that stay together over time may experience increased survival rates [19], a greater potential to breed successfully [19], increased clutch sizes [18] and increased lifetime productivity [7] the longer they stay together. Mate and nest-site fidelity are often linked [8], resulting in pairs remaining together at the same nest site from year to year [4]. Nest-site fidelity, also known as philopatry, occurs when animals remain at, or return to, the same nesting site the next year. Philopatry can result in benefits such as familiarity with a site and its resources [20,21] and avoiding delays in nesting [22]. While moving beyond a nesting territory to a new nest site (breeding dispersal) has the potential to result in lower reproductive success [4], birds may undertake such movements when nesting habitat changes unpredictably [23], or to find higher quality habitat [5] or a higher quality mate [24,25]. Whether to disperse to a new breeding site versus how far to disperse are decisions often affected by different factors [26,27]. Factors known to influence mate fidelity, nest-site fidelity and breeding dispersal distances in avian species include sex [28–30], age [5,6,20,27,31], and breeding success [22,32–34]. Breeding experience with a mate can also affect mate fidelity [6,18] and loss of a mate can influence nest-site fidelity [4].

Many studies have examined differences between urban and non-urban avian populations especially with respect to breeding success and reproductive productivity [35]. And, demographic parameters, such as adult and juvenile survivorship, have been studied in avian populations declining in urban settings [12,36]. But, more study of behavioral responses is needed [2,36], including the potential disruption of mate and nest-site fidelity behaviors in urban populations [4]. Several studies have examined these behaviors in declining populations, but none were in urban settings. For example, Pyle et al. (2001) [7] examined mate fidelity and nest fidelity on breeding success in a Cassin’s auklet population (*Ptychoramphus aleuticus*), breeding on a coastal California island, that was declining due to reductions in ocean food resources. In a declining population of Canada jays (*Perisoreus canadensis*) inhabiting a forested provincial park, Fuirst et al. (2021) [5] predicted breeding distances would decrease as territories became available, but their results showed no change in breeding dispersal distances over time. An analysis of breeding dispersal using banded bird data for 75 species from 1909–1994 in Britain and Ireland did not find dispersal distances were influenced by population trends [37]. Marzluff et al. (2016) [4] studied breeding dispersal and mate fidelity in four species with different tolerances to urban impacts, but this study was not designed to assess these behaviors with respect to population declines.

As urbanization reduces species’ habitats and population sizes, there is a need to quantify within-population processes in order to understand the role these behaviors may play in population declines. Our objective was to investigate mate and nest-site fidelity rates and breeding dispersal distances in relationship to reproductive success in an avian population declining in an urban setting. Given that urban settings can disrupt mate selection and/or the longevity of nesting sites, we suspected that mate and nest-site fidelity rates might change significantly as the study population decreased. Specifically, mate and nest-site fidelity rates might increase and dispersal distances decrease over time as habitat is lost to urban impacts. Disruption of these behaviors could then contribute to the declining population through reduced annual reproductive success over time.

We analyzed mark-resight data for a population of resident western burrowing owls (*Athene cunicularia subspp. hypugaea*) over an 18-year period in Santa Clara County, California, an area of rapid urbanization [38]. The burrowing owl is a short-lived (approximately 5 years) [39], socially-monogamous species of grassland habitats that typically uses burrows of colonial rodents. The western North American subspecies occurs west of the Mississippi River to the Pacific Ocean and from southern Canada to northern Mexico. It is declining throughout its range [40]. The other North American subspecies, *A. c. floridana*, was listed as a threatened species in Florida in 2016 by the state wildlife agency due to habitat loss and other anthropogenic threats [41]. While burrowing owls are known to adapt to some level of human development [42,43], at a point, increasing development pressures can decrease nest densities, nest success rates and brood size [42] as well as reduce population sizes [44]. Several studies have examined burrowing owl mate fidelity, nest-site fidelity and breeding dispersal distances together [26,33,42], but none of these populations were declining in a highly-urbanized environment. By examining mate and site fidelity rates and breeding dispersal distances with respect to annual reproductive success, we sought information on processes that might contribute to population declines in urban populations and factors that might influence these behaviors.

We examined: 1) the relationship between mate fidelity and nest-site fidelity, 2) the influence of year (i.e., the effect of time), bird age, breeding success in the previous year, and years with the same mate on mate fidelity, nest-site fidelity, and dispersal distances, and 3) the effect of mate fidelity, nest-site fidelity, year, age, and years with the same mate on annual reproductive success (Table 1).

**Table 1:** Hypotheses tested, variables included, and statistical tests applied to test each hypothesis.

Null Hypotheses Tested	Response Variables	Explanatory Variables Tested	Statistical Test
1) Relationship between Mate Fidelity and Nest-site Fidelity			
A. Mate and nest-site fidelity will not be related	Number of birds of each sex	Mate fidelity and Nest-site fidelity	Chi-square test
2) Factors affecting Mate Fidelity or Nest-site Fidelity			
A. Mate or nest-site fidelity rates will not differ between males and females	Number of birds of each sex	Mate fidelity and Sex Nest-site fidelity and Sex	Chi-square test Chi-square test
B. Mate or nest-site fidelity rates will not be influenced by year, age, breeding success the previous year or years with the same mate	Mate or nest-site fidelity rates for each sex	Mate fidelity: Year, Age, and Breeding success the previous year Nest-site Fidelity: Year, Age, Breeding success the previous year, Years with same mate	GLMM – Binomial Logistic Regression GLMM – Binomial Logistic Regression
3) Factors affecting Breeding Dispersal Distance			
A. Dispersal distance will not differ between males and females	Distance moved from nest-site of previous year	Sex	GLMM – Gamma (log link)
B. Dispersal distance will not change with year, individual’s age, breeding success the previous year and years with same mate	Distance moved from nest-site of previous year for each sex	Year, Age, Breeding success the previous year, Years with same mate	GLMM – Gamma (log link)
4) Factors affecting Annual Reproductive Success (as measured by number of emergent chicks per brood)			
A. Number of chicks per brood will not be related to mate fidelity or nest-site fidelity	Number of chicks per adult for each sex	Mate fidelity Nest-site fidelity	GLMM – Poisson (log link) GLMM – Poisson (log link)
B. Number of chicks per brood will not be related to year, age, or years with same mate	Number of emergent chicks per adult for each sex	Year, Age, and Years with same mate	GLMM – Poisson (log link)

## Materials and methods

### Study sites

**Fig 1:**
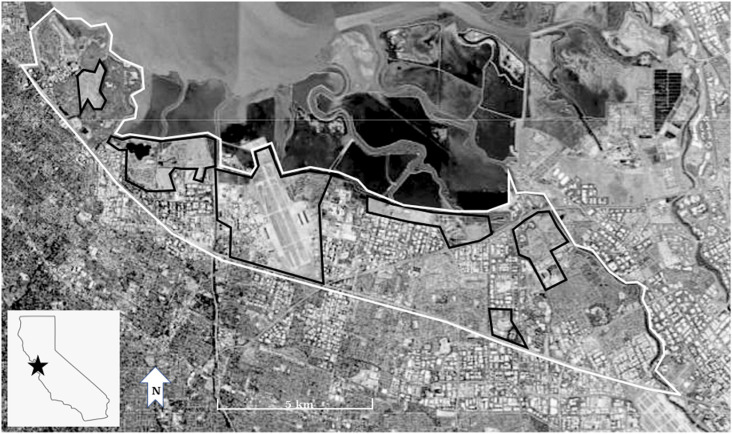
Study region in Santa Clara County, CA (outlined in white) and the breeding burrowing owl study sites (outlined in black), 1999-2016 (Satellite image courtesy of the U.S. Geological Survey).

Banding and nest monitoring occurred during the burrowing owl breeding season, from April 1 through July 31, at breeding sites throughout Santa Clara County, California, USA (Fig 1) from 1999 to 2016, excluding 2006. All sites were located around the south end of the San Francisco Bay in the low-lying valley area of Santa Clara County (Fig 1). The breeding sites consisted of remnant, non-native grassland patches [44] and were bordered by the San Francisco Bay or by dense urban development. Santa Clara County experienced rapid urban development, especially beginning in the 1980s [45], and was estimated to be 80% developed by 2006 with residential, urban and infrastructure uses [38]. The human population density in the urban area was approximately 2,744 people/sq km [38].

From 1999 through 2004, we monitored the population dynamics of breeding burrowing owl colonies at six sites in the study area (Fig 1). We continued to monitor three of these sites from 2005–2012, while three others were periodically monitored (the most westerly site and two most easterly sites in Fig 1). By 2012, the three periodically-monitored sites no longer supported owls; two of them had been lost to urban development and the third site was lost to recreational use that included dogs. From 2012–2016, the three remaining sites were monitored and historic sites were visited, as was an additional, newly managed site for burrowing owls that was within the natal dispersal distance of the closest original site. This new site was not included in the statistical analyses, but was included in the population numbers. Adult population numbers were calculated for 1999–2004 and 2012–2016, the time frames when all extant sites were surveyed.

Walk-through transect surveys were conducted during the day in suitable grassland habitat and at known and historic nest sites to locate potential nests or owls at their burrows [44,46]. Active burrows with whitewash, regurgitated pellets, feathers, bedding material, nest decoration, or prey remains received further observations to determine if a pair was present or if the burrow was occupied by a single bird.

### Nest determination criteria

We undertook field observations from a vehicle at a distance of at least 33 m at dusk, when birds were most active to determine the presence of a pair of owls, detect chick emergence, estimate brood size, and estimate the age of nestlings. A burrow was considered an active nest location if a pair of owls was present or chicks were observed. We observed active burrow locations at least weekly and conducted focused dusk surveys to determine if a pair was present at a burrow. Beginning in early May, we visited and observed nests from a distance, on a weekly basis until chicks were observed or nest failure was determined. We recorded the locations of nests using a hand-held Garmin GPSMap 64 (Garmin, Ltd., Schaffhausen, Switzerland).

### Reproductive status

Breeding success (successful or failed) and annual reproductive success (number of emergent chicks per brood) were determined when emergent chicks that we observed were at least 14 days old [47]. A series of five standardized 30-minute productivity observations, a measure of brood size [48], were performed within seven days after we observed emergent chicks to ascertain brood size and age of nestlings. Observations were separated by an interval of at least six hours. Brood size was determined by the maximum number of ≥14-day old chicks recorded during all observations. A failed nesting attempt was determined by the absence of chicks beyond July 15, when nestlings <14 days old were not reobserved, the death of a female was confirmed, or there was evidence of human or predator disturbance to an active nest location where adults were not reobserved.

### Identification, trapping, and banding

Each breeding season, we banded as many adults and chicks as possible. We resighted bands on previously banded owls with binoculars and a spotting scope. If we were unable to read band codes from a distance, we would attempt to recapture the bird to identify it.

We captured owls using two trapping methods: a 1-way door with bubble trap which captured an owl as it exited the nest/natal burrow or a spring trap (bow-net) employed adjacent to the nest burrow or known foraging site. When we captured an owl, we quickly removed it from the trap, placed it in a sock to keep the owl contained and banded it with a metal bi-colored alphanumeric Acraft band (Acraft Sign and Nameplate Co., Ltd., Edmonton, AB, Canada) (left leg) and a metal U.S. Geological Survey band (right leg). Unbanded males were trapped early in the season. Before capturing an unidentifiable or unbanded female, we monitored the nest to determine her stage in the breeding cycle. Trapping female birds occurred after a successful or failed nesting attempt to eliminate disturbance during fertilization, egg laying, or incubation. Chick trapping and banding occurred after all five 30-minute productivity observations were performed. Typically, chicks were captured when 20–24 days old, prior to dispersing from their natal burrow.

The sex of adults was determined by evaluating sex-specific characteristics, especially the absence or presence of a brood patch, plumage color (females darker in color than males during the breeding season), and behavior. Age was determined by plumage and previous banding history. The age of previously banded chicks (hatch year owls) was definitive. We assigned the age of one year to all newly-banded adults as this is the age at first breeding [7] and natal dispersal distances in other resident burrowing owl populations have been found to be much greater than breeding dispersal distances [30,33,49].

### Statistical analyses

We analyzed 17 years of field data, during an 18-year period from 1999–2016, to identify owls with at least three consecutive years of recorded life history data including sex, age, nest locations, between-season dispersal distances, identity of mates, breeding success, and brood size. For nest-site and mate fidelity analyses, we used only identified females and males for which we were certain the nest attempt was successful or not. Mate fidelity was determined from one season to the next. Owls were considered faithful to a nest site if they nested ≤ 100m from the previous year’s nest site [33]. Dispersal distances between nests were estimated by entering UTM coordinates that we collected in the field into Google Earth Pro©. We then measured the distance moved (m) from one year to the next in Google Earth Pro© to estimate breeding and natal dispersal distances. Annual reproductive success was calculated for nests for which we knew the outcome, either successful or failed.

We conducted all statistical analyses using SPSS (v.28; IBM Corporation) (Table 1). The α-level for significance was 0.05, but we considered levels between 0.05 and 0.2 as approaching significance and providing “evidence of an effect that should be tested with additional studies” [4]. Tests were conducted separately for each sex [7]. Continuous variables included year, age, dispersal distance, years with the same mate, and annual reproductive success as measured by the number of emergent chicks per brood. Categorical variables were mate fidelity (yes or no), nest-site fidelity (yes or no), breeding success the previous year (successful or failed), sex (male or female) and individual bird identification number (Table 1).

To evaluate the relationship between mate and nest-site fidelity as well as overall mate and nest-site fidelity rates by sex, we used Chi-Square tests. Tests met the assumption for Chi-square as all cells had ≥ 5 cases. For all other tests, we used Generalized Linear Mixed Models (GLMM) with individual bird identification as a random variable to address the inclusion of the same birds in multiple years (Table 1). Relationships between mate fidelity or nest-site fidelity and factors that could influence those rates were tested with using a Binary Logistic distribution. The Gamma distribution (log link) was used to compare male to female dispersal distances and for hypotheses testing factors potentially affecting breeding dispersal distances, which was right-skewed, continuous data. Finally, we used the Poisson distribution (log link) to: a) test hypotheses with number of chicks per brood (annual reproductive success), which were count data, b) compare the annual reproductive success of birds that did versus did not show mate or nest-site fidelity and c) assess annual reproductive success with respect to years with the same mate.

### Permits

Trapping and banding were sub-permitted under two United States Geological Survey Federal Bird Banding Permits, one from the Institute for Bird Populations in Point Reyes Station, California (permit number 22423) for 1999–2015 and one from the San Francisco Bay Bird Observatory Coyote Creek Field Station in Milpitas, California (permit number 22109) for 2015–2016. The field research for this study was conducted under two California Department of Fish and Wildlife Scientific Collecting Permits, permit number SC-2220 from 1999–2004 and permit number SC-7012 from 2005–2016. Research was conducted in compliance with San José State University IACUC permit number 795.

## Results

**Fig 2:**
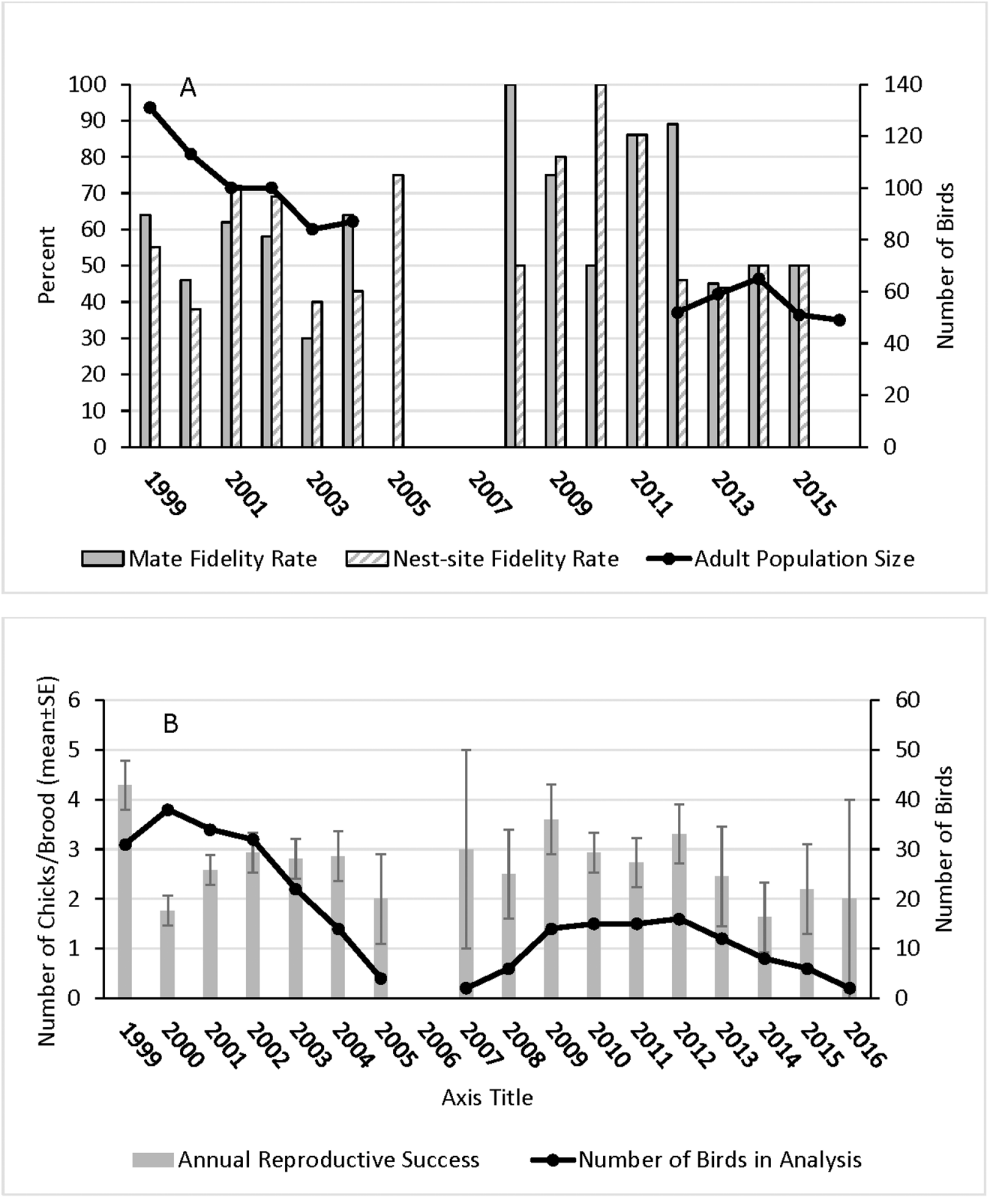
For burrowing owls in the study region in Santa Clara County, CA from 1999–2016: A) mate and nest-site fidelity rates (percent) shown with the overall size of the adult population; B) mean (±SE) annual reproductive success (number of chicks per brood) for successful and failed nests combined, shown with the number of birds included in the analysis.

Adult population numbers for 1999–2004 and 2012–2016 showed a decline within both periods and between the two periods. The population of 131 adults in 1999 dropped to 41 adults by 2016 (Fig 2A), a 69% decline.

For fidelity and dispersal data, we analyzed the records of 65 owls who bred at least two years in a row and for which we knew the identity of the mates, dispersal distances and number of emergent chicks; 36 were females and 29 were males. Of these birds, we also estimated natal dispersal distances for 24 individuals. We did not know the ages of 39 of these 65 owls (22 females, 17 males), as they were captured as adults. We assumed these owls were one-year old when we encountered them as this is the age at first breeding [7]. In addition, natal dispersal distances in our population (median: male = 1,661 m; female = 1,218 m) were much greater than breeding dispersal distances (median: male = 55 m; female = 63 m), a pattern found in other resident burrowing owl populations [30,33,49]. Since natal dispersal distances were much greater than breeding dispersal distances, if owls came from outside our population, they were more likely to be one-year olds than older owls. While there is likely to be some error each year due to these assumptions, we believe the error is small and consistent from year to year. The number of owls included in the study each year ranged from 2–38, most years (11/17) exceeding 10 birds (Fig 2B).

### Relationship between mate fidelity and nest-site fidelity

Mate fidelity and nest-site fidelity were strongly associated with each other overall (*X*^2 ^= 30.917, df = 1, P < 0.001, n = 191) and for each sex (males: *X*^2 ^= 11.350, df = 1, P* *< 0.001, n = 83; females: *X*^2 ^= 19.325, df = 1, P* *< 0.001, n = 108). Of 191 breeding attempts by birds breeding for at least the second year in a row and for which the mate was known, 82 (42.9%) were at the same nest-site with the same mate the next year. When birds moved, they were likely to be paired with a different mate from the previous year (51/191 = 27.7% of breeding attempts), versus being with the last year’s mate (24/191 = 12.6%). And, birds showing nest fidelity were paired with a new mate in only 16.8% (32/191) of breeding attempts.

### Mate fidelity and nest-site fidelity rates and factors

Of 191 breeding attempts by 65 birds breeding for at least the second year and for which the mate was known, 55.5% of pairs remained together. Mate fidelity rates did not differ by sex (males = 59.0% and females = 52.8%) (*X*^2 ^= 0.744, df = 1, P = 0.388, n = 191). Year, i.e., the effect of time, was not a significant factor for either males or females (Table 2); mate fidelity rates fluctuated over the study period, but not in relation to population change (Fig 2A). Age was not a factor in mate fidelity for males, while this factor was important for females (Table 2); in particular, females at age 3 and 4 were more likely to stay with mates (25/33 = 75.8% and 12/20 = 60.0% of breeding attempts, respectively) than females at age 2 (16/30 = 53.3%). Breeding success the previous year was a very important factor for males; males who successfully reproduced stayed with mates the next year in 85.1% (40/47) of breeding attempts.

**Table 2:** Factors potentially affecting mate fidelity, nest-site fidelity, breeding dispersal distances and annual reproductive success for burrowing owls during the study period, 1999–2016, in the Santa Clara County, CA study area. Significant results in bold. Results approaching significance in italics.

	Year		Age		Breeding Success the Previous Year^*^		Years with Same Mate	
	F-value; P-value	*B* (±SE); 95% CI	F-value; P-value	*B* (±SE); 95% CI	F-value; P-value	*B* (±SE); 95% CI	F-value; P-value	*B* (±SE); 95% CI
*Mate Fidelity*								
Males (df = 1,76)	0.164; 0.686	−0.022 (0.054); −0.130–0.086	0.211; 0.647	0.110 (0.240); −0.367–0.588	**8.013; 0.006**	**1.596 (0.564); 0.473**–**2.719**	na^**^	
Females (df = 1,100)	0.793; 0.375	−0.039 (0.044); −0.127–1.941	**5.417; 0.022**	**0.432 (0.186); 0.064 - 0.800**	*2.364; 0.127*	*0.848 (0.551);* *−0.246–1.941*	na^**^	
*Nest-Site Fidelity*								
Males (df = 1,74)	0.405; 0.527	−0.038 (0.060); −0.157–0.081	1.478; 0.228	−0.327 (0.269); −0.864–0.209	1.171; 0.283	0.660 (0.610); −0.556–1.876	**7.471;** **0.008**	**−1.047 (0.383);** **−1.811** – **−0.284**
Females (df = 1,99)	0.006; 0.936	0.004 (0.055); −0.105–0.113	*3.547;* *0.063*	*0.390 (0.207);* *−0.021–0.802*	*2.061;* *0.154*	*0.875 (0.609);* *−0.334–2.084*	**9.242;** **0.003**	**−0.919 (0.302);** **−1.518** – **−0.319**
*Breeding Dispersal Distance*								
Males (df = 1,82)	1.139; 0.289	0.035 (0.033); −0.030–0.100	1.584; 0.212	−0.188 (0.150); −0.486–0.110	0.044; 0.833	0.074 (0.351); −0.624–0.772	**57.475;** **<0.001**	**−1.205 (0.159);** **−1.521** – **−0.888**
Females (df = 1,101)	0.631; 0.429	0.044 (0.055); −0.065–0.153	*3.795;* *0.054*	*0.250 (0.128);* *−0.005–0.505*	**10.640;** **0.002**	**1.218 (0.374);** **0.477–1.959**	**78.361;** **<0.001**	**−1.492 (0.169);** **−1.826** – **−1.158**
*Annual Reproductive Success*								
Males (df = 1,101)	0.017; 0.896	0.005 (0.039); −0.072–0.083	0.012; 0.913	−0.015 (0.136); −0.284–0.255	na^**^		**6.913;** **0.010**	**0.562 (0.214);** **0.138–0.987**
Females (df = 1,134)	0.188; 0.665	−0.017 (0.040); −0.097–0.062	*3.096;* *0.081*	*−0.180 (0.102);* *−0.383–0.022*	na^**^		**11.184;** **0.001**	**0.592 (0.177);** **0.242–0.943**

* Reference category was “Successful” (=0) breeding in the previous year.

** Factor was correlated with the response variable.

**Fig 3:**
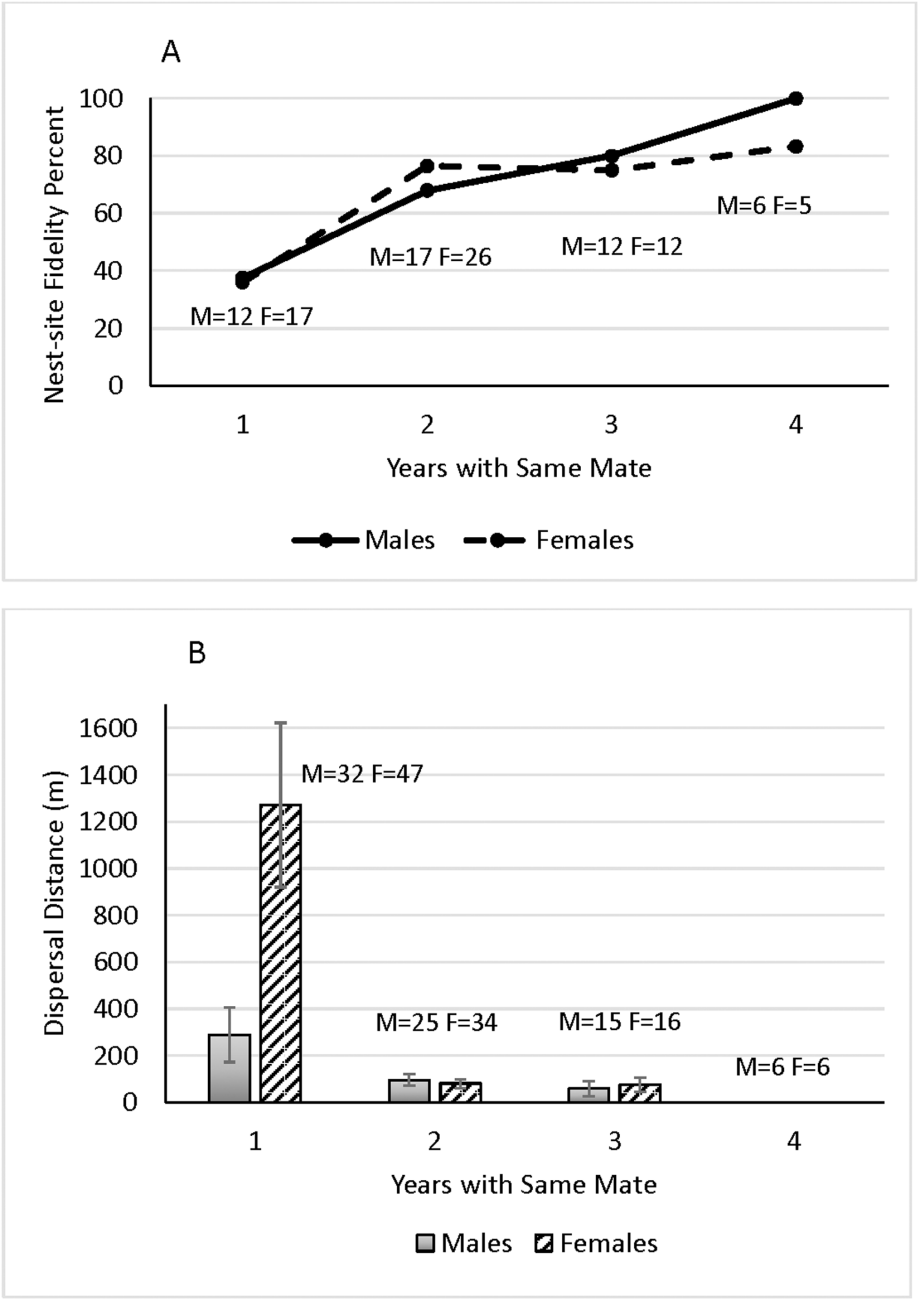
Relationship between years with the same mate and A) nest site fidelity rates (percent) and B) mean (± SE) breeding dispersal distances (m) for male and female burrowing owls during the study period, 1999–2016, in the Santa Clara County, CA study area. Number of birds of each sex shown at points or bars.

Of 199 breeding attempts by 65 burrowing owls breeding for at least the second year and for which nest-site fidelity was known, 59.7% of owls remained at their nest site the next year. Site fidelity rates did not differ by sex (males = 61.4% and females = 55.9%) (*X*^2 ^= 0.612, df = 1, P* *= 0.434, n = 199). Year was not an important factor in this parameter for either males or females (Table 2), as nest-site fidelity rates fluctuated over time (Fig 2A). Nor was age related to nest-site fidelity for either sex, although age approached significance for females. Breeding success the previous year was not a significant factor in nest-site fidelity for either sex, although it approached significance for females. But, for both males and females, years with the same mate was strongly, positively related to nest-site fidelity (Table 2, Fig 3A).

### Breeding dispersal distance

Of 199 breeding attempts by 65 burrowing owls breeding for at least the second year and for which dispersal distance was known, males dispersed an average of 187.6 ± 52.0 m (median = 54.6 m, n = 88) and females 623.8 ± 158.5 m (median = 62.8 m, n = 111), which was a significant difference in breeding dispersal distance by sex (F(1,216) = 6.533, P = 0.011). Most owls stayed within 100 m of the previous year’s nest site, but a few owls of each sex—especially females—dispersed relatively long distances. The longest distance a male dispersed was approximately 2,000 m, while eight females dispersed between approximately 2,000 and 10,000 m.

Neither year nor age were significant influences on dispersal distances for either sex, although age approached significance for females (Table 2). Breeding success the previous year was an important factor for females, who dispersed a median of 42 m when breeding was successful versus 213 m when not. While breeding success was not statistically significant with respect to distance dispersed for males, those who were successful breeders dispersed a median of 31 m compared to 137 m for males who were not successful. The number of years birds spent with the same mate strongly influenced dispersal distances for males and females. Specifically, birds dispersed further after spending only one year with a mate compared to distances dispersed when they spent more years with that mate (Table 2, Fig 3B).

### Annual reproductive success

**Fig 4:**
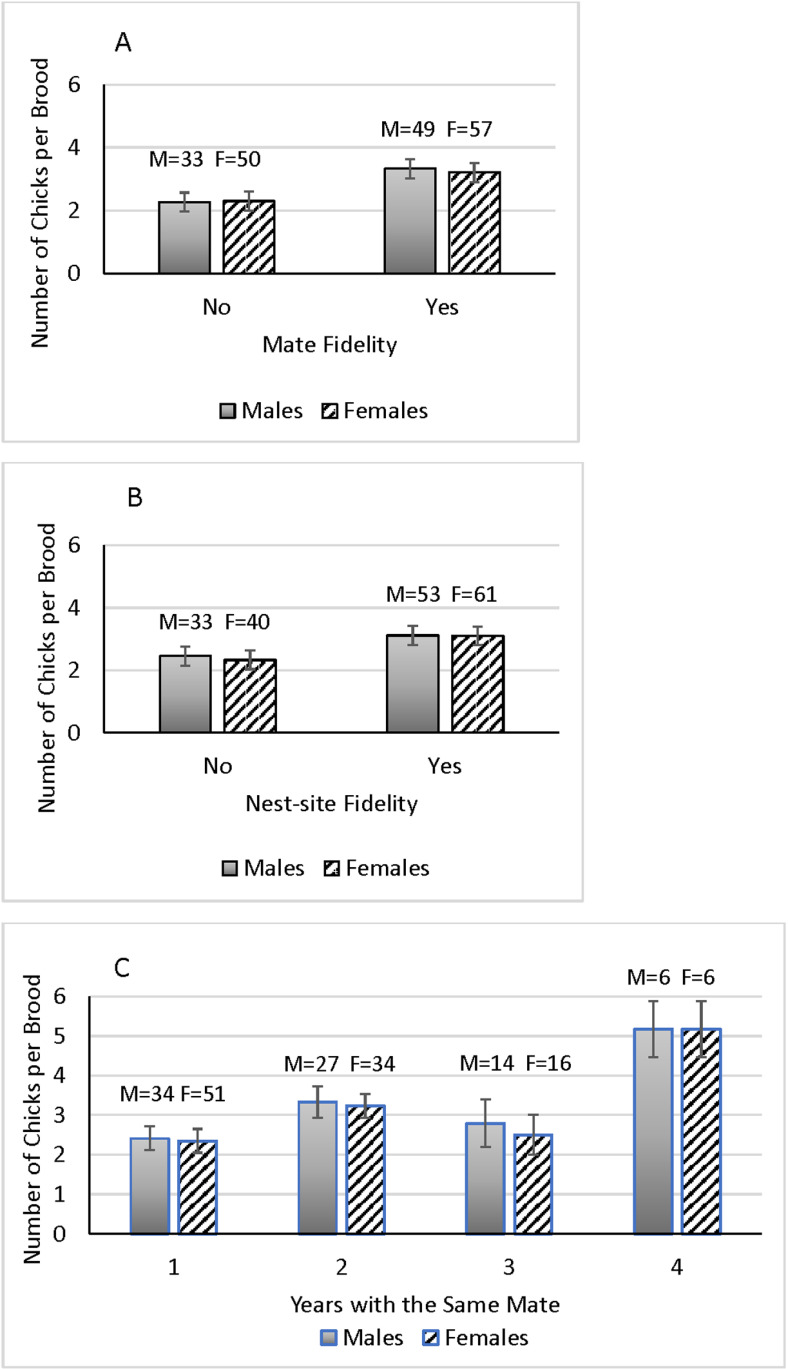
Influence of A) mate fidelity, B) nest-site fidelity, and C) number of years with the same mate on annual reproductive rate, as measured by the mean (± SE) number of chicks per brood for male and female burrowing owls during the study period, 1999–2016, in the Santa Clara County, CA study area. Numbers of birds per sex shown above bars.

Both males and females who stayed with the same mates the next year produced more chicks per brood than birds who switched mates (males: F(1,78) = 6.612, P = 0.015; females: F(1,102) = 5.610, P = 0.020) (Fig 4A). While not significant, average brood sizes were 22% larger (2.38 versus 3.06 chicks) for males displaying nest-site fidelity compared to those who dispersed (F(1,81) = 1.288, P = 0.230). Average brood size was larger by 24% (2.33 versus 3.05 chicks) for females who stayed at the previous year’s nest site versus females who dispersed (F(1,105) = 3.832, P = 0.053) (Fig 4B). Annual reproductive success rates did not differ between any year, except potentially 1999 and 2000 which seemed to differ from each other and all other years (Fig 2B). Year and age were not factors in annual reproductive success for either males or females (although age approached significance for females), but years with the same mate was important for both sexes (Table 2). The longer owls stayed with the same mate, the greater the number of chicks they tended to produce (Fig 4C).

## Discussion

Urban development is causing the decline of avian populations worldwide. This study provided insight into whether the disruption of mate fidelity, nest-site fidelity or breeding dispersal distance might contribute to population declines. We examined these behaviors in a population of urban burrowing owls that declined by 69% over the course of an 18-year period and found no change in the fidelity behaviors, breeding dispersal distances or annual reproductive success over time. These results differed from our prediction that mate and nest-site fidelity rates might change as the population decreased and that these disrupted behaviors might result in reduced annual reproductive success over time. In this urban-adaptable species, mate and nest-site fidelity rates were maintained in the face of urban pressures.

The population we studied exhibited fidelity behaviors typical of many avian species, including: 1) mate and nest-site fidelity were strongly associated in both sexes [7,8]; 2) neither sex dispersed far when they moved, although a few birds, especially females, moved quite long distances [27,37]; and 3) when dispersal occurred, females moved further than males [5,6]. In addition, breeding success the previous year was an important factor in mate fidelity for male owls, as has been found in previous studies of birds [7,33], and males that were successful breeders dispersed shorter distances than those that were not successful. While this factor was marginally important to females with respect to mate and nest-site fidelity, breeding success the previous year for females resulted in shorter dispersal distances than for birds that were not successful.

Although burrowing owls are relatively short-lived, we found age was an important factor for females with respect to mate fidelity and potentially for nest-site fidelity, dispersal distance and annual reproductive success. The age of birds has been related to increasing reproductive output in some species, especially in long-lived birds [7]. In addition, we found that years with the same mate—a longevity-related factor—was strongly related to producing more offspring over time for both sexes. That is, as pairs remained together, they tended to produce more chicks and, therefore, would be expected to improve lifetime reproductive success over switching mates. While staying with a mate has been shown to improve reproductive success in long-lived bird species [7,50], our findings indicate that, even in shorter-lived species, staying with a mate longer can improve reproductive success, a finding that adds to our understanding of the factors affecting these fidelity behaviors in short-lived bird species [18].

In this study population, more offspring were produced when mates stayed together and showed nest-site fidelity compared to when pairs split or moved the next breeding season. Marzluff et al. (2016) [4] found that nest site fidelity can have significant advantages over moving nesting locations, even when habitat conditions are not optimal, as in disturbed urban conditions. However, Stow and Sunnocks (2004) [16] suggest that fragmented populations may exhibit detrimentally high mate and nest-site fidelity rates compared to non-urban populations, behaviors which could ultimately reduce reproductive success [14]. We found the 56% mate fidelity rate in our population was higher than the rates in two resident burrowing owl populations for which there was data—a growing Florida population that inhabited an urbanizing residential area (approximately 273 people per km^2^ in 1990) [30] and a slightly declining southern California population in an agricultural setting [33,46]. The mate fidelity rates for these populations were 47% and 42%, respectively. Also, the median dispersal distances moved by our owls were half to one quarter the dispersal distances found for the two other resident burrowing owl populations [27,30]. These differences in mate retention and dispersal distances may have been due to the isolation of our population from other burrowing owls [51], as the birds in our study were confined to discrete, relatively small habitat patches compared to the other two populations. It is possible that birds may have dispersed farther than we could detect and bred elsewhere but, as recorded in other bird species [37], very few burrowing owls have been found to disperse great distances between breeding attempts [49]. However, examining the potential for longer-distance dispersal and determining the fate of dispersing owls are topics for further study.

The nest-site fidelity rate in our population, at 60%, was somewhat lower than the other two populations (Florida: 78%; southern California: 66%). Whether the mate fidelity, nest-site fidelity and dispersal distances we observed were resulting in a “fidelity-induced ecological trap” [17] is not clear. However, the number of chicks produced annually per pair in our population did not decline over time, which may indicate these behaviors were not reducing annual reproductive fitness. That said, annual and lifetime reproductive success as well as other fitness measures for our study population would need to be compared with stable populations not living in urban habitat conditions in order to understand the potential for such an ecological trap.

There is increasing evidence that even urban-adaptable species, such as the burrowing owl, are not necessarily protected from population declines in the face of mounting urban pressures [52]. Beyond examining the role of changes in fidelity behaviors, this study was not designed to determine other potential causes of the decline of this urban population. However, certainly, one contributor was the loss of three of the six occupied sites, extant at the start of the study, to urban development and impacts. While a new site came under management for burrowing owls during the last five years of the study, the number of owls there could not compensate for previous habitat losses and the continued decrease in numbers at the remaining original sites. To understand the causes of the decline, future research could examine potential contributing factors such as the extent of land use change in the study area, changes in adult and juvenile survivorship, changes in predator density over time, adequacy of prey, rates of soil surface disruption and/or human recreational disturbance [43–45].

These findings showed that, in species with linked mate and nest-site fidelity, long-term protection of nest-sites is one key to maintaining these fidelity behaviors and promoting pair longevity, which can result in increased reproductive success. Conditions which promote adult survivorship will also contribute to the ability of pairs to remain together. For managers, this may mean taking measures to reduce factors that cause birds to lose partners, as we found that starting a new breeding relationship resulted in fewer offspring than if birds stayed with their original partners.

Despite these measures, urban sites may impose pressures that could ultimately cause such habitats to be population sinks [17]. Burrowing owls are an urban-adaptable species that can maintain important fidelity behaviors in human-altered habitats. However, even such species are subject to population declines in urban settings. To support healthy breeding populations of burrowing owls and other species with similar breeding behaviors, it is essential to protect and establish populations in locations with extensive, protected, stable, and high-quality habitat that allows extended mate and nest-site fidelity.
